# Multidisciplinary approaches to the Amazonian past: introduction to the theme issue

**DOI:** 10.1098/rsfs.2022.0068

**Published:** 2022-12-09

**Authors:** Nicholas Q. Emlen, Leonardo Arias, Rik van Gijn

**Affiliations:** ^1^ Leiden University Centre for Linguistics, Leiden, The Netherlands; ^2^ University of Groningen (Campus Fryslân), The Netherlands; ^3^ Department of Evolutionary Genetics, Max-Planck-Institute for Evolutionary Anthropology, Leipzig, Germany

**Keywords:** Amazonia, genetics, linguistics, ethnography, archaeology, history

## Abstract

This theme issue presents collaborative research by anthropologists, linguists, archaeologists, geneticists, historians and biogeographers, who work across disciplinary boundaries to investigate the Amazonian past. Amazonia is a fertile ground in which to develop such multidisciplinary approaches because its relative paucity of documentary records makes other sources of evidence regarding the past more important; because multidisciplinary approaches are well suited to address important unanswered questions in Amazonian history; and because a recent and dramatic reappraisal of the region's past make this an exciting time to conduct this sort of research. The papers in this theme issue feature different combinations of academic disciplines, and they address different geographical regions and historical periods, but all of them show how combining insights from different fields can help illuminate aspects of the Amazonian past that would otherwise remain obscure to them all.

This theme issue of *Interface Focus* brings together the work of researchers who investigate the Amazonian past from a range of disciplinary perspectives. The issue grew out of a workshop held during the 2021 conference of the Society for the Anthropology of Lowland South America (SALSA), hosted by the University of Virginia, which featured collaborations among anthropologists, linguists, archaeologists, geneticists, historians and biogeographers. The challenge that these contributors have sought to address is a familiar one in the contemporary research landscape: each of those disciplines has made great strides in illuminating their respective aspects of the Amazonian past, but the increasing specialization and technical sophistication of the disciplines, and the volume of empirical data that they generate, has made it more difficult than ever to work across the disciplines to build an integrated historical picture. In light of this challenge, each of the contributions to this theme issue attempts the hard work of bringing together the data, methods, or theories of more than one discipline to clarify a specific aspect of the Amazonian past. The complexity of this endeavour is reflected in the long list of authors who have contributed their diverse disciplinary expertise to these papers.

The papers address various geographical regions of Amazonia, which is a fertile ground in which to develop such multidisciplinary approaches. For one, Amazonia is characterized by a relative paucity of documentary records, which makes other sources of evidence regarding the past more important. Furthermore, South America remains today the historically ‘least known continent’ [1]—the Amazonian region, in particular—with data from fields like genetics, linguistic typology and archaeology representing a tiny fraction of their coverage in other regions of the world. In this context, working together across disciplines is important for interpreting the findings from any one of them, and also holds the promise of revealing more about the past than is possible in other, more intensively studied regions. Finally, the scholarly understanding of the Amazonian past has undergone a dramatic reappraisal in the last generation or two, with a new consensus that the region was not always characterized by small-scale societies in a so-called ‘counterfeit paradise’ which constrained their land use practices and size [2], but rather that it has also been home to societies of notable sociopolitical complexity and scale, engaging in intensive food production and large-scale landscape modification (e.g. [3]). These developments raise new questions relevant to the full range of academic disciplines listed above, making this a particularly exciting moment in the study of the Amazonian past. Moreover, the expanding availability of data across several of the relevant disciplines has increased the potential strength and validity of such approaches.

The papers in this theme issue vary in their geographical and temporal focus (though there is considerable overlap among them). Two papers examine the historical dynamics of the linguistically diverse Northwest Amazonian region around the Upper Rio Negro in Colombia and Brazil (Chacon & Cayón [4]; Arias *et al*. [5]), while van Gijn *et al*. [6] take a broader geographical perspective on the same region, extending the scope to Ecuador and Northern Peru. Zariquiey *et al*. [7] examine the Panoan language family, which is located further south in the Amazonian regions of central Peru, northern Bolivia and western Brazil. The paper by Michael *et al*. [8] focuses on the Arawakan family, which is present in all of those areas, extending across much of the Amazon Basin and further north into the Guyanas and the Caribbean. Regarding timescales, the papers that examine the Northwest Amazon (van Gijn *et al*. [6], Cayón & Chacon [4], Arias *et al*. [5]) take a similar scope: these authors focus on Arawakan–Tukanoan contact, which likely emerged during the last 2500 years and continues to the present day [9]. Michael *et al*.'s [8] study of the Arawakan language family includes archaeological dates ranging from 2800 BP to 500 BP. Zariquiey *et al*. [7] do not mention dates, but the Panoan family has been called ‘shallow’ [10], with estimates of its diversification ranging widely from a possible 3000 years [11] to about 1000 years BP [12].

The paper by van Gijn *et al*. [6] asks what mechanisms might have given rise to the unexpectedly high degree of linguistic genealogical diversity in some areas of Amazonia, paying special attention to the Northwest Amazon (this diversity is unexpected in light of the relatively recent peopling of South America). The paper contrasts two mechanisms of linguistic diversity maintenance that have been proposed in the literature: through relative isolation, and through a system of conscious identity preservation in the context of contact and interaction. These mechanisms make contrasting predictions about the historical traces that we would expect to find in the geographical, ethnographic, genetic and linguistic records. van Gijn *et al*. [6] set out to test these predictions in the Northwest Amazon using data from these four disciplines, and they conclude that the region was characterized by a period of shared history between many groups, over large distances, until around 500 CE. Around that time, groups practicing intensive agriculture expanded throughout the Northwest Amazon. The ensuing period featured the emergence of a close relationship between speakers of Arawakan and Eastern Tukanoan languages, as well as generally less intensive interactions between those groups and the speakers of languages from smaller families. A more detailed look at some of the smaller language families and isolates, moreover, reveals considerable differences in their social dynamics, ranging from relative isolation to full integration in the emerging regional system.

The second paper in the theme issue, by Cayón & Chacon [4], also explores the history of the social and linguistic dynamics of the Northwest Amazon, though through a different methodological approach and using a narrower geographical and linguistic scope. The authors ask how the linguistic and cultural diversity of the Upper Rio Negro emerged in a situation of intense contact relations between the groups of the area, focusing on the Tukanoan, Arawakan and Nadahupan language families. Their analysis integrates the historical linguistics of those families with the region's ethnographic and archaeological records, to understand how and when the various language groups came to interact within the Upper Rio Negro linguistic and social system as it developed over the course of centuries and millennia. In this manner, they propose a detailed, multi-layered reconstruction of the region's history, which takes into account both the movement of populations and the emergence of a multi-ethnic social structure featuring stable multilingualism and the maintenance of linguistic differences as organizational principles.

The third paper, by Arias *et al*. [5], zooms in even further on the same region to investigate the extensive population contact between the speakers of Yukuna (an Arawakan language) and Tanimuka (an Eastern Tukanoan language), and the impact of that contact on the genetics of the two ethnolinguistic groups and their linguistic structures. These authors test two hypothetical historical scenarios to explain the observed patterns of variation: first, that the ancestors of today's Tanimuka speakers spoke an Arawakan language related to Yukuna, and later shifted to Eastern Tukanoan; or second, that the ancestors of today's Tanimuka speakers spoke an Eastern Tukanoan language, and that the genetic similarities between the groups are due to intermarriage that also resulted in linguistic convergence between the two languages. The authors consider these scenarios using data from genetics, linguistics and ethnography, and conclude that both shift and convergence were likely to have operated during the long relationship between those two groups. Because the distinctive indigenous social structure of the Northwest Amazon was created through the interaction between speakers of Arawakan and Eastern Tukanoan languages, this case study informs the region's social and linguistic dynamics more generally.

The fourth paper, by Michael *et al*. [8], also examines the history of the Arawakan language family, and like the paper by Cayón & Chacon [4], it brings together archaeological and historical linguistic evidence. The authors take steps toward clarifying one of the most important problems in South American linguistic history: the internal structure of the Arawakan language family, and the time depth of its expansion and diversification into sub-branches. The authors note that the latter issue has remained unclear in part because time estimates of the family's diversification have been based on the mistaken assumption of constant rates of change; they argue that what is needed instead are estimates that allow for variable rates of change. Such estimates require calibration, however, and given the lack of documentation for ancient South American languages, archaeological materials associated with the Arawakan expansion represent an important source for such calibrations. Michael *et al*. [8] thus propose a set of archaeological calibration points and connect them to Arawakan linguistic subgroups in space and time, allowing for different degrees of uncertainty. These calibration points can be used to more precisely pinpoint particular nodes of an Arawakan phylogenetic classification in time, thus allowing for a more reliable application of an analysis with a variable change rate assumption. This integration of historical linguistic and archaeological data thus promises to clarify the time depth and internal structure of the Arawakan family, a key set of issues in our understanding of the Amazonian past.

Finally, like the previous paper, Zariquiey *et al*. [7] also focus on a single language family: Panoan. The authors combine quantitative methods with more traditional historical linguistic analysis to illuminate the development of Panoan body part expressions, which are mostly combinations of bound body part roots and morphologically opaque formatives. The bound roots and the formatives show different diachronic patterns: while the body part roots tend to be conservative and reconstructible in the protolanguage, the formatives behave unpredictably, in some cases reflecting old patterns, and in other cases resulting from recent innovations. By bringing these phenomena together, the authors show how a narrow lexical domain can offer a rich source of information regarding the historical development of a language family. In addition to the paper's implications for our understanding of the Panoan language family, it also contributes to the long-standing debate about differential rates of change in linguistics—a point that is essential for integrating linguistics with other disciplines to reconstruct the past. This paper also underscores the importance of combining quantitative methods with the knowledge of experts who work closely on particular language families.

Within the pages of this theme issue, the many contributing authors have reached beyond their own disciplinary expertise and attempted to reconcile their own data and analyses with those of their co-authors. These collaborations have required not only immersion in the technical aspects of other research fields, but also engagement with the markedly different perspectives and assumptions about human experience that underlie each one. Joint progress in such a context is hard-won, but the papers in this theme issue show how combining insights from different disciplines can help illuminate aspects of the Amazonian past that would otherwise remain obscure to them all.

## Data Availability

This article has no additional data.
